# Testicular Germ Cell Tumors: Paraneoplastic Syndromes and the Role of Beta-Human Chorionic Gonadotropin

**DOI:** 10.7759/cureus.14286

**Published:** 2021-04-04

**Authors:** Toufic Tannous, John Miskovsky, Matthew Keating

**Affiliations:** 1 Internal Medicine, Roger Williams Medical Center/Boston University, Providence, USA; 2 Department of Medicine, Roger Williams Medical Center, Providence, USA; 3 Division of Hematology and Oncology, University of California Irvine, Irvine, USA

**Keywords:** choriocarcinoma syndrome, testicular germ cell tumor, paraneoplastic hyperthyroidism

## Abstract

Choriocarcinoma syndrome is a rare phenomenon that occurs in male patients with testicular choriocarcinoma. Male patients who have a testicular non-seminomatous germ cell tumor (TNSGCT) with at least partial choriocarcinoma histology, and metastases to the lungs and/or other extragonadal sites, as well as a markedly elevated beta-human chorionic gonadotropin (HCG), have been prone to pulmonary bleeding, hypoxia, and acute respiratory distress syndrome (ARDS). The respiratory complications occur immediately after chemotherapy is administered or, in some cases, spontaneously. Paraneoplastic hyperthyroidism is another entity described in patients with testicular choriocarcinoma, whereby high levels of HCG (typically >50,000 mIU/ml) induce clinical and laboratory characteristics of hyperthyroidism. We present the case of a male patient diagnosed with TNSGCT and found to have both choriocarcinoma syndrome and paraneoplastic hyperthyroidism in the setting of only mildly elevated HCG levels. We compare our case with similar cases published previously while questioning the quantitative role of HCG.

## Introduction

Testicular tumors, especially germ cell tumors (GCT), are the most common malignancies affecting male patients between the ages of 15-35 years old. GCT can be either seminomatous or non-seminomatous. Non-seminomatous GCT include yolk sac tumors, choriocarcinoma (CC), embryonal, teratoma, or a combination thereof. The mixed type composes 30-50% of all GCT [1]. Although treatment is widely available and most patients with a non-seminomatous germ cell tumor (NSGCT) have chemosensitive disease with high cure rates, the choriocarcinoma type is an extremely malignant subset prone to brain, liver, and lung metastases with a five-year overall survival less than 80% [2]. CC is composed of several types of cells including cytotrophoblasts, intermediate trophoblasts and syncytiotrophoblasts. These cell types are known to secrete beta-human chorionic gonadotropin (HCG) which in high levels contributes to certain presenting symptoms such as gynecomastia and hyperthyroidism. These types of tumors are prone to bleeding and necrosis due to their highly vascular and rapidly proliferating nature which causes them to outgrow their blood supply [3]. In fact, hemorrhage is considered to be the cause of death in around 44% of testicular CC cases [4].

Choriocarcinoma syndrome (CS) occurs in patients with choriocarcinoma GCT and diffuse metastases, especially to the lungs, presenting with pulmonary hemorrhage and markedly elevated HCG levels. A comprehensive review by Rejlekova et al. describes high-risk patients for CS with testicular non-seminomatous germ cell tumors (TNSGCT) as having lung metastasis, CC pathology and high levels of HCG (especially above 50,000 mIU/ml) [5]. Identification of high-risk choriocarcinoma patients is extremely important as chemotherapy delivered early can improve survival. The wait for a formal tissue biopsy diagnosis may delay treatment. Therefore, a young male presenting with a testicular mass, lung metastasis and elevated levels of HCG > 50,000 mIU/ml should trigger prompt treatment. At the same time, clinicians need to be cautious when considering chemotherapy regimens, as treatment can induce CS and thus reductions in the treatment regimen have been proposed as a solution to reduce the risk [6,7].

In addition to CS, hyperthyroidism is a paraneoplastic syndrome that is well-described in CC patients and linked to elevated HCG levels. It is suspected that in most cases with high levels of HCG > 50,000 mIU/ml, patients are prone to developing a hyperthyroid state. The alpha subunit of the HCG hormone is similar to that of thyroid stimulating hormone (TSH) and thus could actually stimulate the TSH receptor and lead to hyperthyroidism or even thyrotoxicosis. Furthermore, the association of elevated HCG and hyperthyroidism extends to female patients with invasive moles, gestational choriocarcinoma, and placental site trophoblastic tumors.

We present a unique case of CS with acute respiratory distress syndrome (ARDS) and paraneoplastic hyperthyroidism in a young male with TNSGCT and a mildly elevated HCG level. We then compare our findings to similar cases published in the literature.

## Case presentation

**Figure 1:**
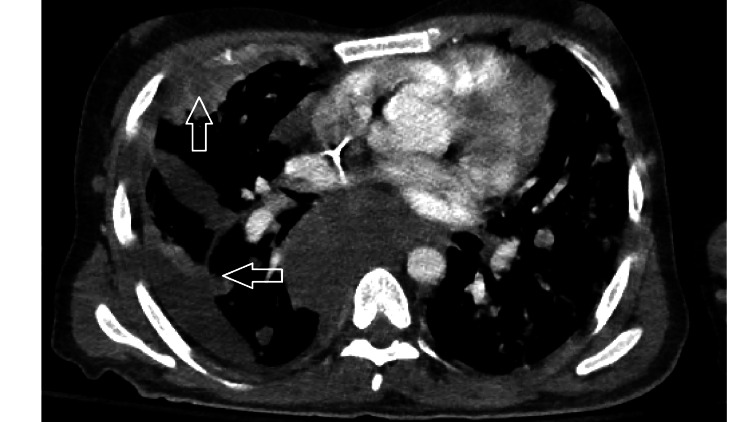
Pulmonary nodules and surrounding edema as seen on chest CT on day 2 of chemotherapy.

**Figure 2:**
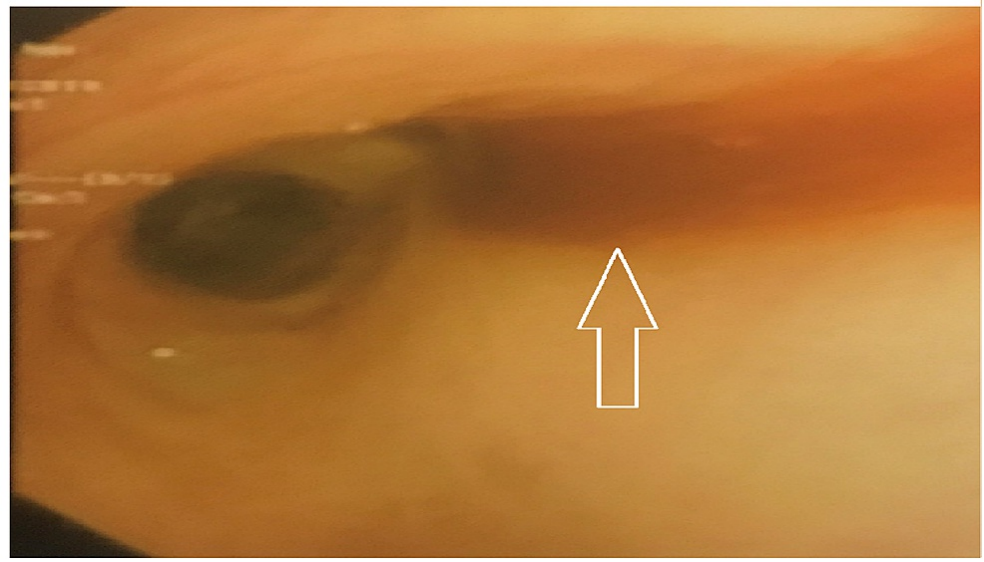
Blood seen in the right main stem bronchus during bronchoscopy performed after initiation of chemotherapy.

**Figure 3:**
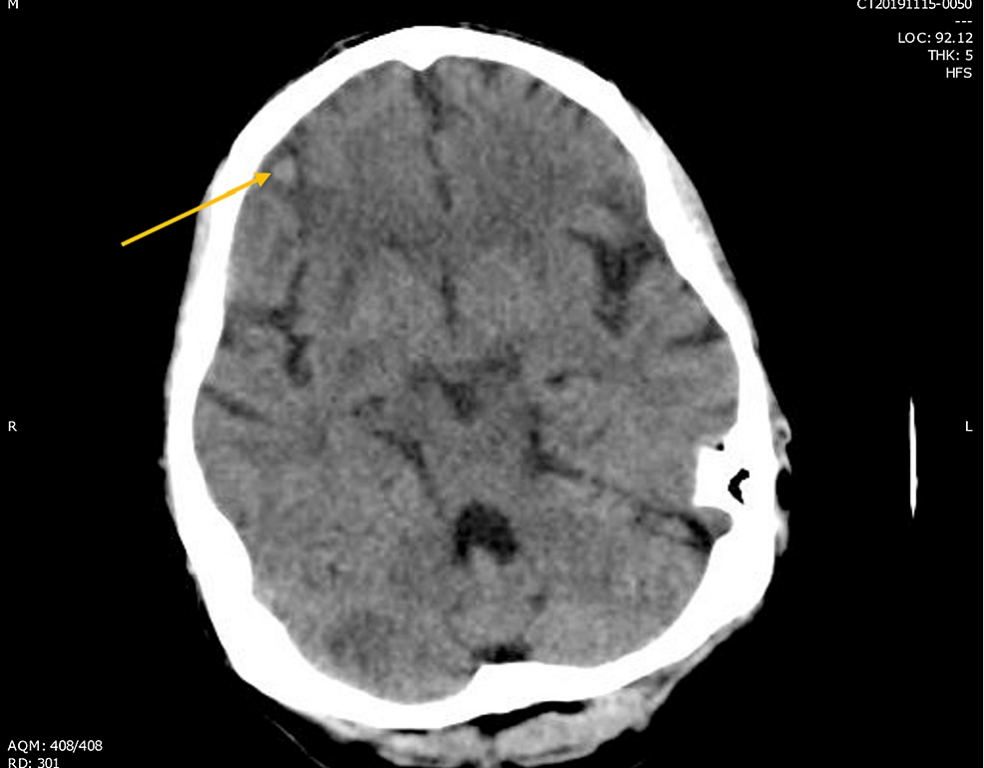
Arrow showing suspected cerebral hemorrhage on CT head.

**Figure 4:**
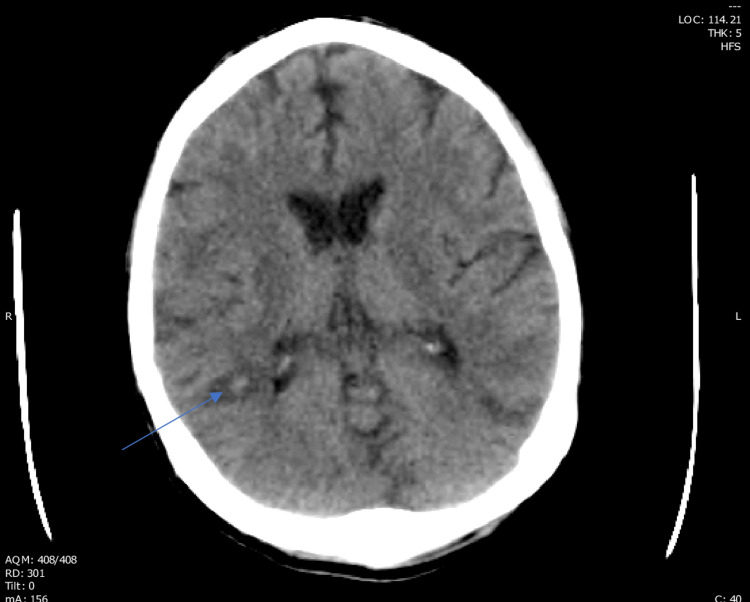
Arrow showing suspected cerebral hemorrhage surrounding the 6 mm tumor on CT head.

A previously healthy 29-year-old Caucasian male presented with two weeks of worsening dyspnea and one month of scrotal swelling. He had no prior smoking or drinking history and did not use recreational drugs. His family history was non-contributory. Upon presentation to the emergency room, his physical exam revealed tachycardia, decreased breath sounds at the right lung base, marked scrotal and lower extremity edema, and a right testicular mass. Labs on admission included an HCG level of 1,063 mIU/ml (normal 0.5 - 5 mIU/ml), alpha fetoprotein level (AFP) of 26,157 ng/ml (normal 0 - 9 ng/ml), lactate dehydrogenase level (LDH) of 1,671 U/L (normal 140 - 271 U/L), TSH level < 0.01 uIU/ml (normal 0.34 - 5.6 uIU/ml), and a free thyroxine level (T4) of 3.1 ng/dl (normal 0.6 - 1.7 ng/dl). Computed tomography (CT) with contrast of the chest, abdomen and pelvis revealed multiple pulmonary masses, right pleural effusion, extensive bulky mediastinal and abdominal adenopathy, hepatic lesions, and an 11.7 cm right testicular mass. The patient underwent right radical orchiectomy and tumor resection, with pathology revealing high-grade mixed germ cell tumor composed of choriocarcinoma (50%), yolk sac tumor (45%) and embryonal carcinoma (5%). At that point he was diagnosed with a high-grade metastatic mixed germ cell tumor with metastases to the lungs and liver. Given the high burden of lung metastases, traditional BEP (bleomycin, etoposide, and cisplatin) chemotherapy was deferred in favor of VIP (etoposide, ifosfamide and cisplatin) chemotherapy. During his hospital stay he complained of persistent headaches, and a brain magnetic resonance imaging (MRI) showed multiple small brain lesions with the biggest measuring six millimeters in the right temporo-parietal region. He started the first cycle of his induction chemotherapy as an inpatient with VIP, as well as methimazole and propranolol for hyperthyroidism. On day two of chemotherapy, he became acutely hypoxemic and developed ARDS requiring intubation with intensive care unit (ICU) transfer. Chest CT demonstrated new ground-glass opacities surrounding the pulmonary nodules (Figure 1), and bronchoscopy confirmed diffuse alveolar hemorrhage (Figure 2). He finished his first cycle of chemotherapy while intubated, and the pulmonary hemorrhage resolved over the following days. His course was complicated by rapidly increasing numbers of hemorrhagic brain metastases (Figures 3, 4), renal failure requiring hemodialysis, pancytopenia, sepsis, and acute stroke. Due to his poor prognosis and quality of life, he chose to be terminally extubated 29 days after admission.

## Discussion

The first two index cases of CS were published by Logothetis in 1984 [8]. In describing these cases, he emphasized the unique clinical behavior of choriocarcinoma relative to other histologic subtypes of testicular cancer. While acknowledging the significance of HCG level in terms of representing tumor volume and overall prognosis, his description of choriocarcinoma syndrome revolved around the choriocarcinoma histology and pulmonary hemorrhagic complications rather than a set HCG cutoff. This phenomenon was initially described in the pretreatment setting, independent of chemotherapy administration, and was dubbed CS. Thereafter, many other cases have been reported and published in the literature, both prior to and precipitated by chemotherapy. We focused our search on CS and ARDS cases that developed shortly after chemotherapy as with our patient.

CS can occur in the setting of a gonadal or extra-gonadal primary tumor. The suspected pathophysiology is presumed to be a massive tumor lysis syndrome and cytokine release, often precipitated by chemotherapy in certain high-risk groups. This cytokine storm may eventually lead to diffuse alveolar hemorrhage (DAH) and ARDS [9]. Due to pulmonary bleeding and hypoxia, there is a high mortality rate in these cases. The current standard of treatment for poor-risk metastatic non-seminomatous germ cell tumors consists of four cycles of chemotherapy, either bleomycin, etoposide, and cisplatin (BEP) or etoposide, ifosfamide and cisplatin (VIP). The HCG tumor marker is monitored closely to assess response. Usually in high burden disease presenting with metastasis, etoposide and cisplatin (EP) are given initially. Bleomycin is given later to avoid potential respiratory failure. If bleomycin cannot be tolerated, or there is significant preexisting lung disease or renal failure or advanced age over 50, an ifosfamide-based regimen is given instead, typically VIP.

Using the search engine PubMed, case series and case reports documenting CS and/or ARDS developing shortly after chemotherapy were collected and summarized in Table 1. All these cases described CS and/or ARDS in male patients diagnosed with testicular germ cell tumors and metastases with a predilection for the lungs, liver, and/or brain. The predominant histology was choriocarcinoma. Most but not all HCG levels were above 50,000 mIU/ml, and all patients received chemotherapy resulting in CS and/or ARDS. The mortality rate was extremely high and only a few survived after developing ARDS. The criteria used by all authors to label this complication as CS were based on the clinical presentation: metastatic TNSGCT, elevated HCG often over 50,000 mIU/ml, evidence of bleeding at sites of metastasis, and development of hypoxia due to ARDS/DAH immediately after chemotherapy. It seems that elevated levels of HCG above 50,000 mIU/ml represented a common theme for most of these cases. However, whether this indicates causation or is merely an associated finding remains unclear.

**Table 1:** Chemotherapy-induced CS and/or ARDS cases over the past four decades. CS: choriocarcinoma syndrome; ARDS: acute respiratory distress syndrome; CC: choriocarcinoma; HCG: beta-human chorionic gonadotropin; BEP: bleomycin, etoposide, and cisplatin; VIP: etoposide, ifosfamide and cisplatin

Author & year	Cases (n)	Histology	CS and/or ARDS	Chemo regimen	HCG	Mortality (%)
Kirch et al. 2003 [7]	16	6 CC, 5 embryonal, 2 undetermined, 1 mixed, 1 yolk sac + teratoma, 1 embryonal + teratoma	ARDS	Various regimens	HCG 81,400-413,000 IU/l in CC; normal-276,000 IU/l in other cases	9/16 (56.3)
Massard et al. 2010 [9]	16	Not specified	ARDS	Various regimens	HCG 11-8,920,000 UI/l	12/16 (75)
Moran-Ribon et al. 1994 [13]	11	6 CC, 2 embryonal, 2 CC + teratocarcinoma, 1 yolk sac + teratocarcinoma	ARDS (and hemoptysis reported in 4 patients)	Various regimens	HCG <10 mIU/ml in 1 case; HCG 50-8,920,000 mIU/ml in 3 cases; HCG 9,114-1,080,000 mIU/ml in 7 cases	11/11 (100)
Kandori et al. 2010 [14]	1	Yolk sac	CS + ARDS	BEP	HCG 630,000 mIU/ml	0/1 (0)
Kobatake et al. 2015 [15]	1	CC + yolk sac	CS + ARDS	BEP	HCG 150,670 mIU/mL	1/1 (100)
Zeitjian et al. 2019 [10]	1	CC	CS + ARDS	VIP	HCG 274,465 IU/l	1/1 (100)
Kobayashi et al. 2019 [16]	1	CC	CS + ARDS	BEP	HCG 822,290 mIU/ml	0/1 (0)
Motzer et al. 1987 [17]	2	1 CC + embryonal, 1 CC	CS	Carboplatin; Cisplatin/Vinblastine/Bleomycin	HCG 1,670 ng/ml; HCG 38,000 ng/ml	1/2 (50)
Tatokoro et al. 2008 [18]	1	Yolk sac + immature teratoma	CS	Etoposide/Ifosfamide/Cisplatin	HCG 534,000 mIU/mL	0/1 (0)
Kawai et al. 2006 [19]	1	CC + seminoma	CS	BEP	HCG 2,660,000 IU/ml	0/1 (0)
Arana et al. 2012 [20]	1	CC	CS	BEP	HCG >200,000 IU/l	0/1 (0)

The identification of CS and ARDS has evolved with time. As can be seen in Table 1, more cases of choriocarcinoma syndrome have been reported, particularly over the past two decades, as the phenomenon has gained wider acceptance and awareness. Chemotherapies can certainly cause ARDS in the absence of CS. But CS has come to be understood as one of many causes of ARDS. We suspect that a few of the reported testicular cancer cases of chemotherapy-induced ARDS may have also had underlying CS. A few reported cases did make this relationship clear, and we denoted this accordingly in Table 1. But in cases where CS is not confirmed by bronchoscopy or imaging evidence of bleeding at metastatic sites, this is difficult to prove.

The HCG level remains highly significant for testicular cancer prognostication and treatment. Post-orchiectomy HCG levels are key determinants of both non-seminomatous testicular cancer staging and risk group stratification. There are well-defined, evidence-based HCG thresholds of prognostic significance, including HCG > 50,000 mIU/ml which delineates the poor risk subgroup. Additionally, tumor markers including HCG hold such importance in testicular cancer that their levels are directly incorporated into testicular cancer staging, thereby extending far beyond the treatment-monitoring role assigned to tumor markers in other cancers. In extenuating circumstances, a markedly elevated HCG level in a male patient with a testicular mass prompts initiation of chemotherapy even without waiting for histologic confirmation of testicular cancer.

The quantitative significance of the HCG level with respect to CS risk is not well-defined. Due in part to the rarity of CS, there are no evidence-based thresholds for HCG level in relation to CS risk. There are general trends that appear to be present in the reported cases, with a clear tendency toward markedly higher HCG levels in CS. This poses the question of whether the risk of CS can be mitigated both before and after chemotherapy treatment in a patient who has an especially high HCG level at presentation. It has been suggested to modify one’s treatment regimen for cases of elevated HCG with pure choriocarcinoma histology and widespread lung disease [6]. One can reduce the chemotherapy dose which in theory may reduce the incidence of CS [10]. As we have found in our literature search, CS is not always limited to high threshold HCG levels, and CS is by no means limited to pure choriocarcinoma histology. Massard et al. have theorized that a large burden of choriocarcinoma on the lungs, as with our patient, more directly impacts the pathophysiology of CS risk than the HCG hormone itself, which makes sense given that choriocarcinoma in particular is prone to angiogenesis [7]. But an especially high HCG level even in the absence of CC histology should heighten one’s estimation of CS risk. McKendrick et al. reported that half of their patients with markedly elevated HCG levels did not have CC histology identified on initial biopsy [11]. We found a few published cases in our search who went on to develop CS under these circumstances of non-choriocarcinoma histology, as well as several cases like our own with CS and mixed histology.

Our patient had a similar presentation to the cases described in the reports in Table 1. He presented with shortness of breath and a testicular mass. He was found to have a high-grade TSGCT with pulmonary, liver and (later) brain involvement. Surprisingly, his HCG level was 1,063 mIU/ml, a relatively low level in such cases, despite the high-grade nature of his disease as well as his high tumor burden. On day two of his VIP regimen, he developed hypoxia and ARDS. At this point we began to suspect CS. To further confirm our suspicion, a bronchoscopy showed evidence of DAH (Figures 1, 2) and a head CT showed cerebral bleeding. While using a first-line chemotherapy regimen intended for poor-risk patients (VIP), we unfortunately caused our patient to develop CS and ARDS.

Another surprising finding was the low TSH measured on admission. Most cases of TNSGCT that present with hyperthyroidism usually have a markedly elevated HCG [12]. In this situation his HCG was quite lower than what one might expect and yet he presented with laboratory evidence of hyperthyroidism. Our case was unique as it involved two rare complications of TNSGCT, CS and paraneoplastic hyperthyroidism, that are usually seen with extremely elevated levels of HCG. This made us question the quantitative importance of the hormone and shift our focus more towards its qualitative value in paraneoplastic syndromes.

## Conclusions

CS is an extremely lethal complication that may occur post chemotherapy in high-risk patients with TNSGCT of pure or mixed histology and even mildly elevated levels of HCG. Despite using the chemotherapy regimens recommended for this high-risk group, CS may still occur. Clinical and laboratory evidence are well-defined for the staging and risk stratification of testicular CC; however, more robust studies are required to define high-risk groups for CS and paraneoplastic hyperthyroidism.
